# Supplementary material for: Discovery of 12O—A Novel Oral Multi-Kinase Inhibitor for the Treatment of Solid Tumor
Source: Molecules. 2020 Nov 9;25(21):5199. doi: 10.3390/molecules25215199 (PMC7664879; doi:10.3390/molecules25215199)

Supporting Information

Novel oral multi-kinase inhibitor of CDKs and FLT3 for treatment of solid tumor

Yan Fan [1,#], Zhi Huang [1,#] , Xiaoshuang Wang [1], Yakun Ma [1], Yongtao Li [1], Shengyong Yang [2] and Yi Shi [1,*]

Table of Content

Figure S1. Strategy of designing novel inhibitors. 4

Figure S2. The inhibition curve of 12O against CDKs and FLTs. 5

Figure S3. 12O effectively inhibited the proliferation of SKOV3 cells as determined by colony formation assay. 6

Figure S4. ^{1}H and ^{13}C spectra of compound 12A. 7

Figure S5. ^{1}H and ^{13}C spectra of compound 12B. 8

Figure S6. ^{1}H and ^{13}C spectra of compound 12C. 9

Figure S7. ^{1}H and ^{13}C spectra of compound 12D. 10

Figure S8. ^{1}H and ^{13}C spectra of compound 12E. 11

Figure S9. ^{1}H and ^{13}C spectra of compound 12F. 12

Figure S10. ^{1}H and ^{13}C spectra of compound 12G. 13

Figure S11. ^{1}H and ^{13}C spectra of compound 12H. 14

Figure S12. ^{1}H and ^{13}C spectra of compound 12I. 15

Figure S13. ^{1}H and ^{13}C spectra of compound 12J. 16

Figure S14. ^{1}H and ^{13}C spectra of compound 12K. 17

Figure S15. ^{1}H and ^{13}C spectra of compound 12L. 18

Figure S16. ^{1}H and ^{13}C spectra of compound 12M. 19

Figure S17. ^{1}H and ^{13}C spectra of compound 12N. 20

Figure S18. ^{1}H and ^{13}C spectra of compound 12O. 21

Figure S19. ^{1}H and ^{13}C spectra of compound 12P. 22

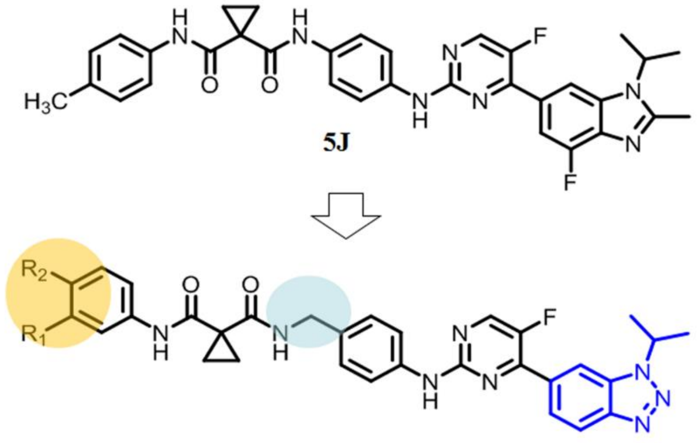

Figure S1. Strategy of designing novel inhibitors.

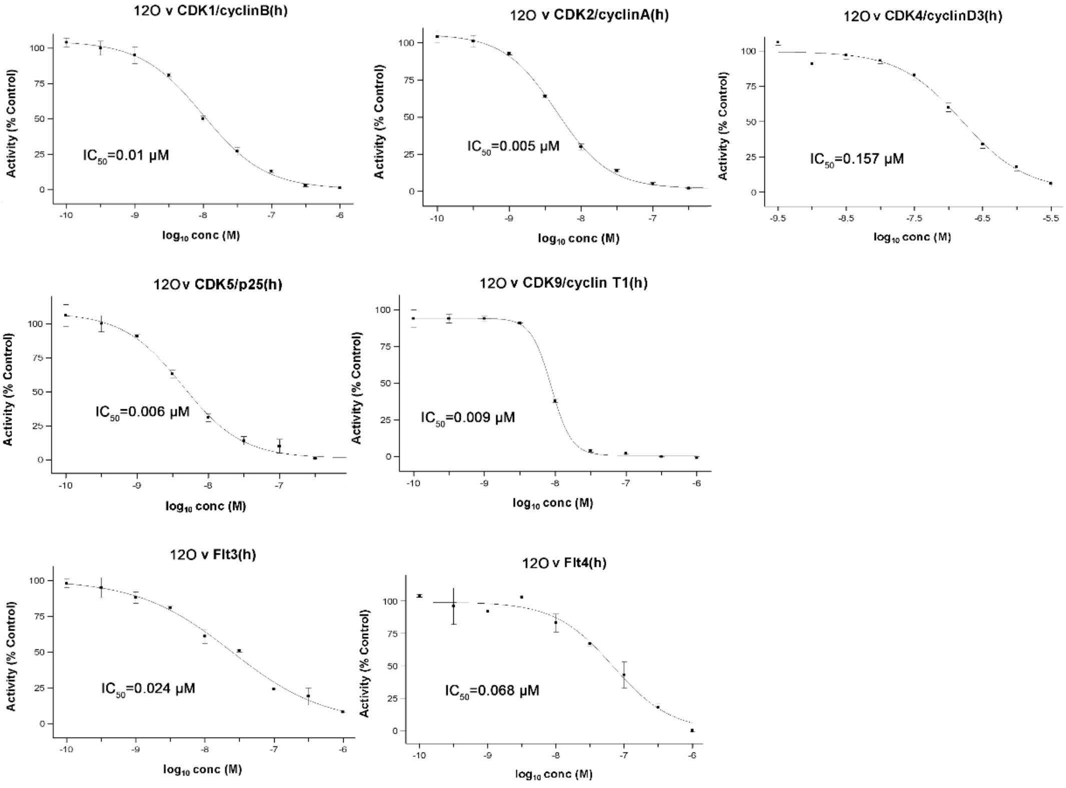

Figure S2. The inhibition curve of 12O against CDKs and FLTs.

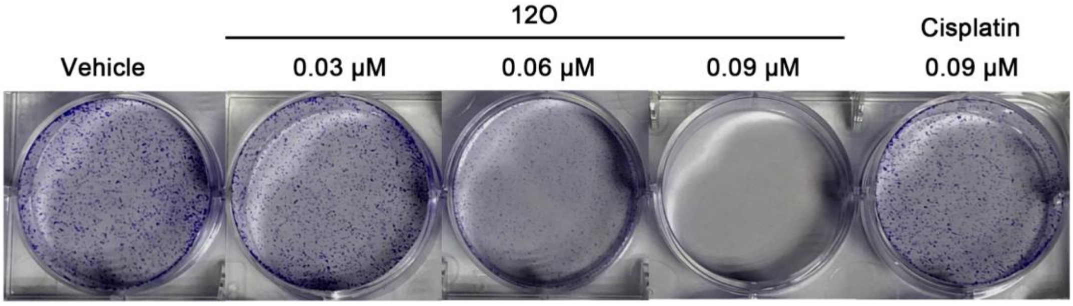

Figure S3. 12O effectively inhibited the proliferation of SKOV3 cells as determined by colony formation assay.

Figure S4. ^{1}H and ^{13}C spectra of compound 12A.

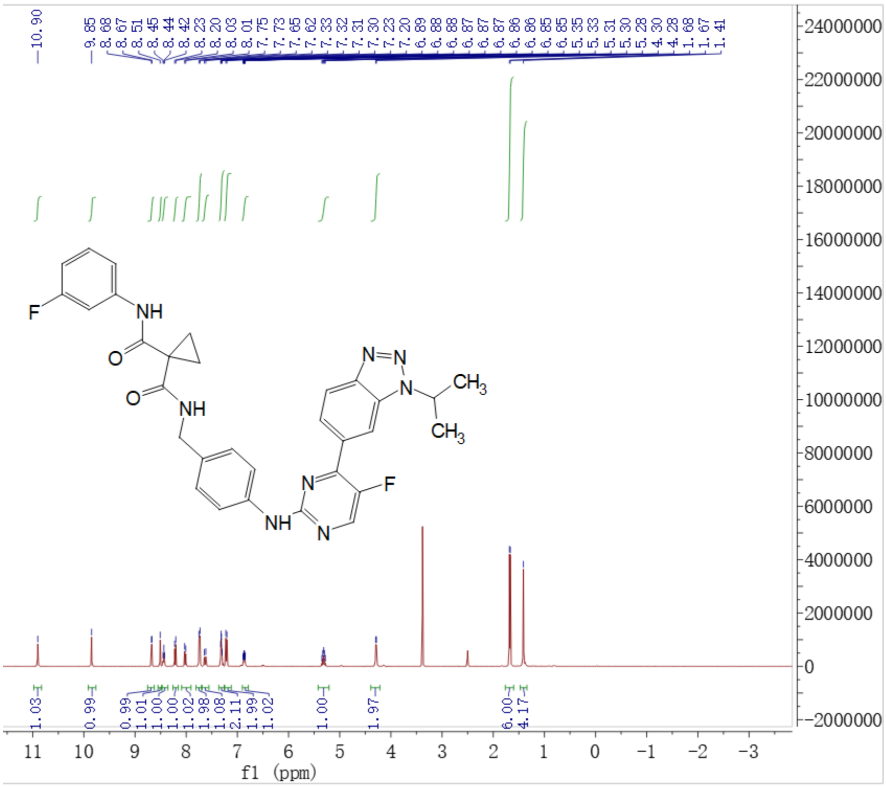

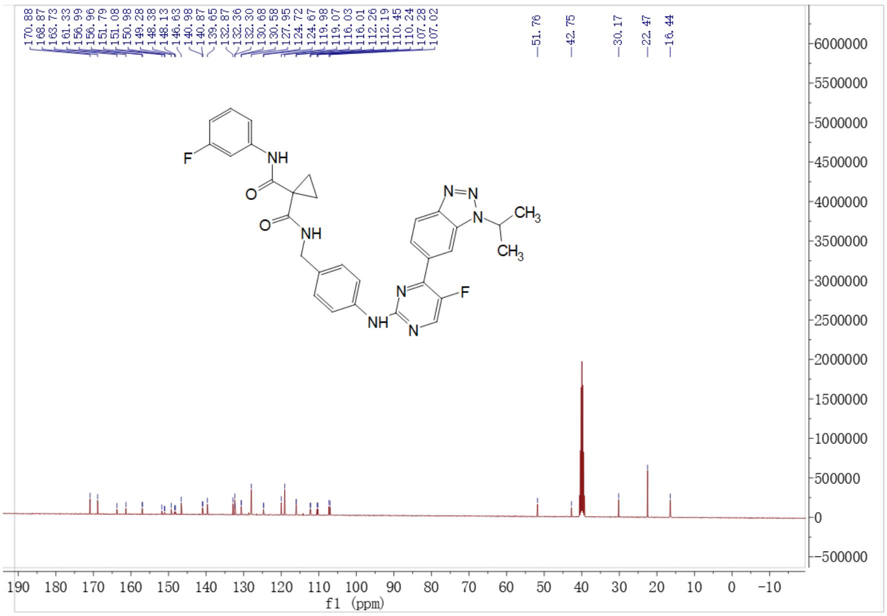

Figure S5. ^{1}H and ^{13}C spectra of compound 12B.

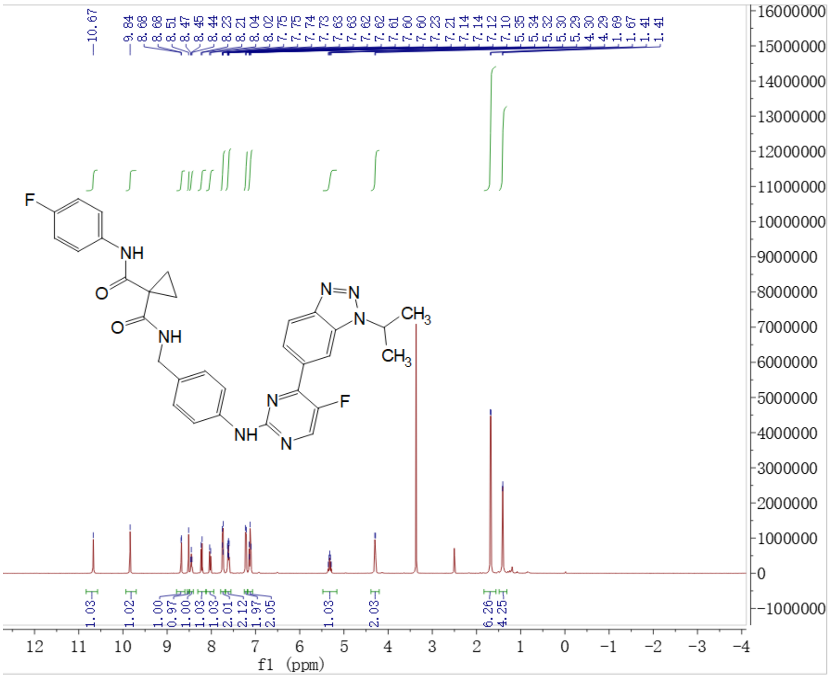

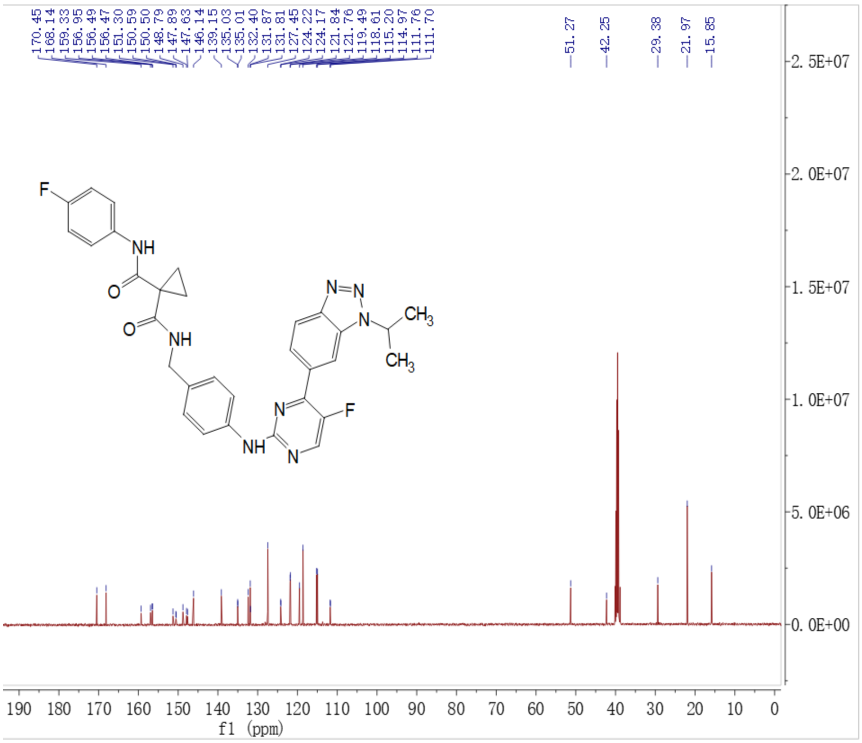

Figure S6. ^{1}H and ^{13}C spectra of compound 12C.

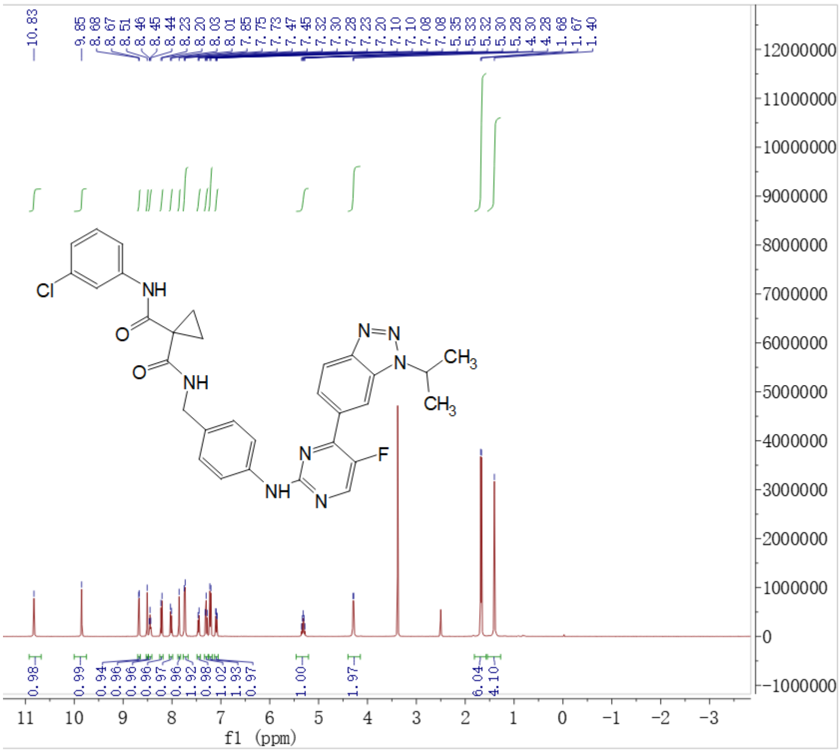

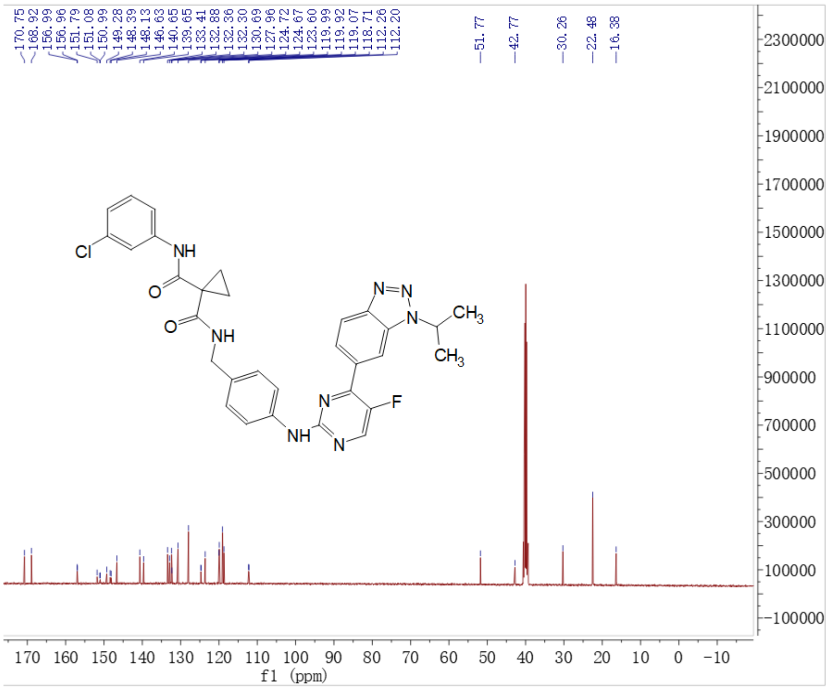

Figure S7. ^{1}H and ^{13}C spectra of compound 12D.

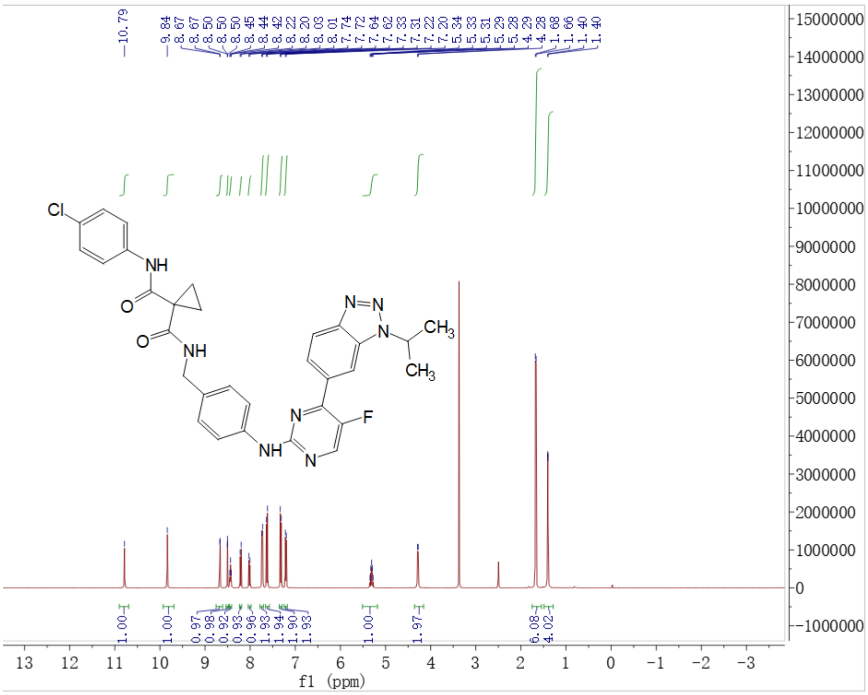

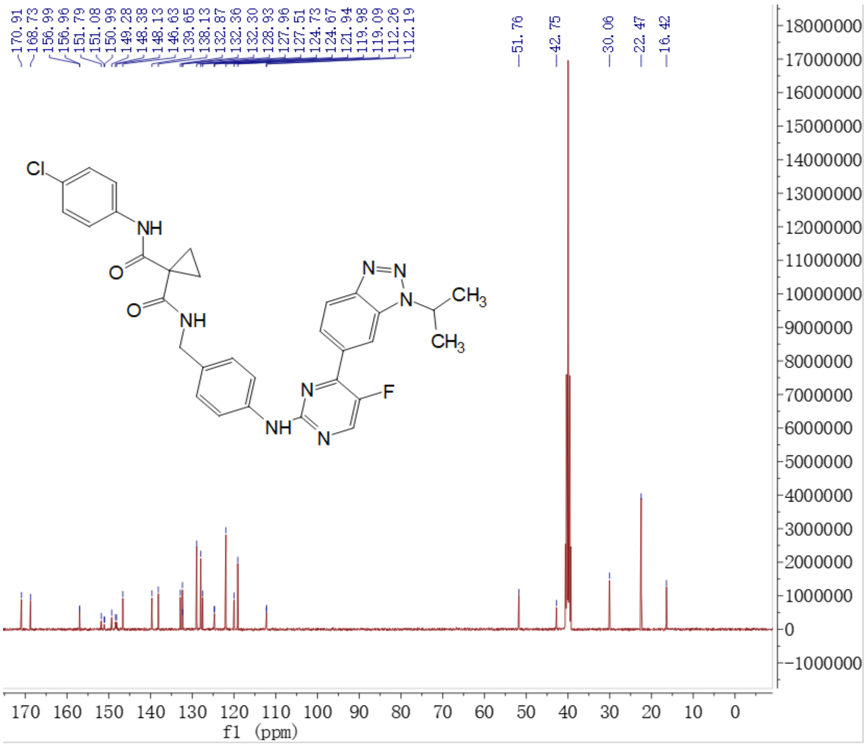

Figure S8. ^{1}H and ^{13}C spectra of compound 12E.

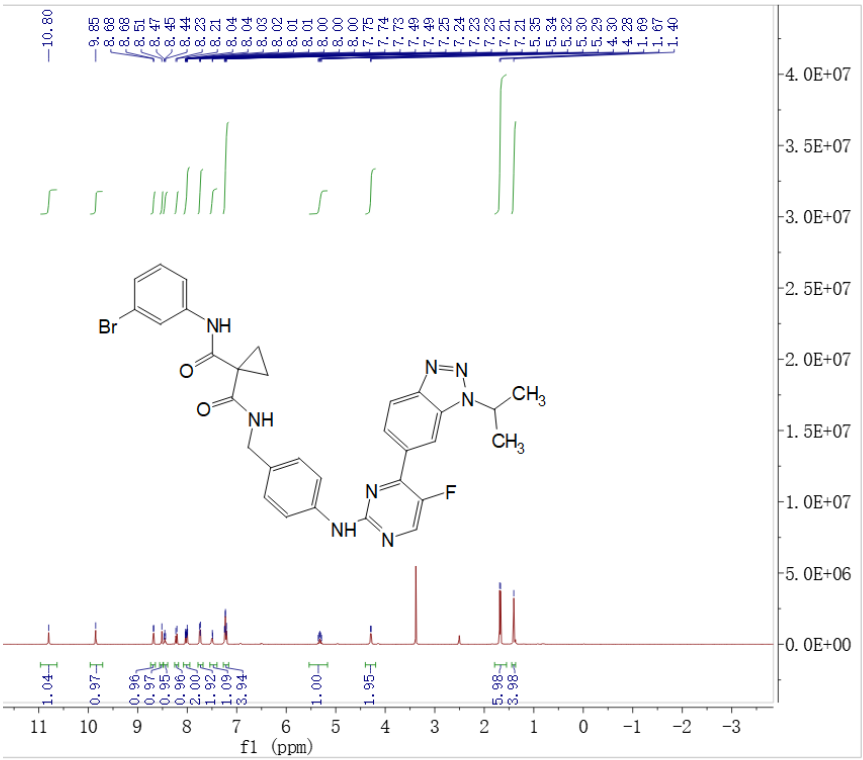

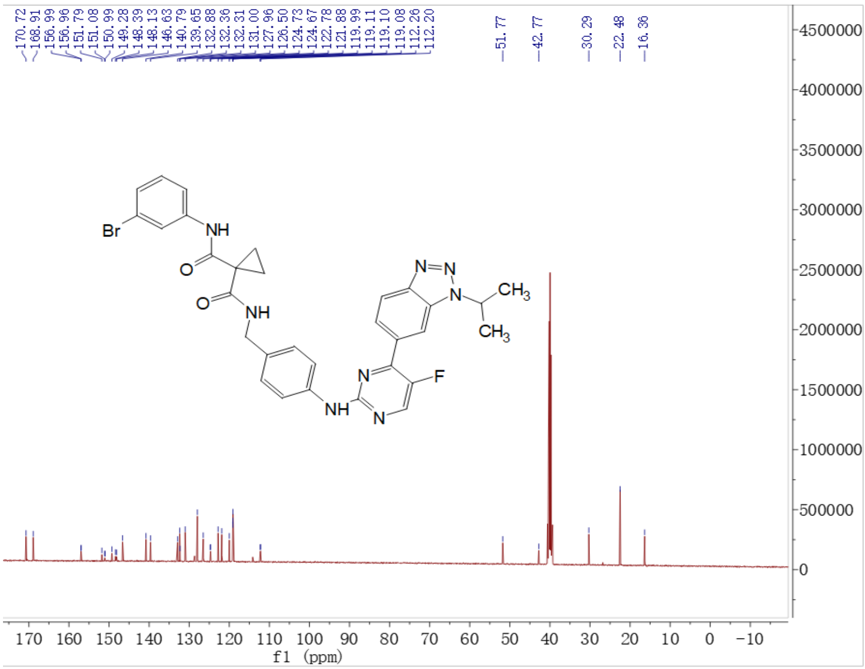

Figure S9. ^{1}H and ^{13}C spectra of compound 12F.

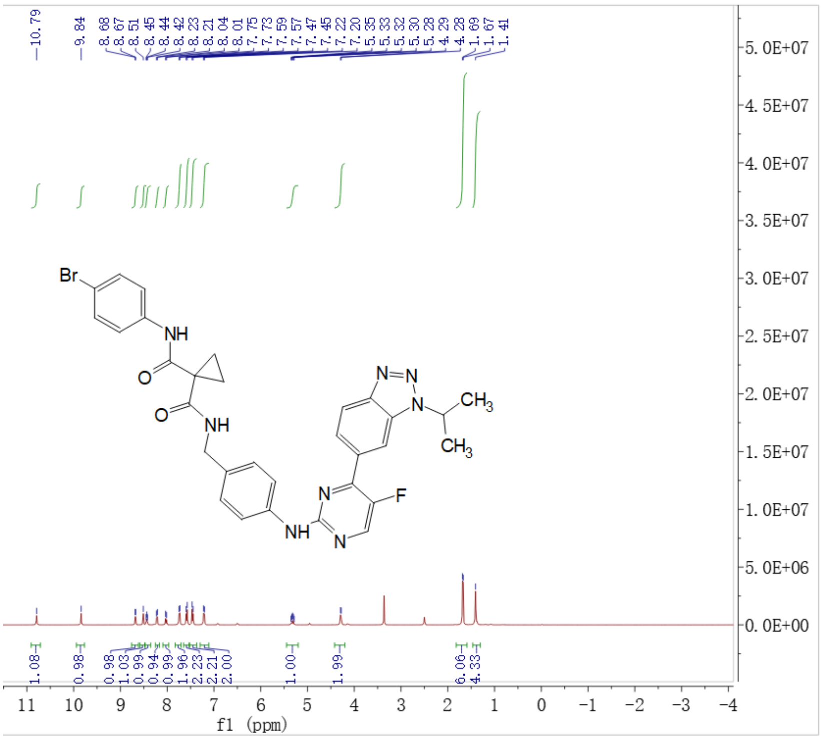

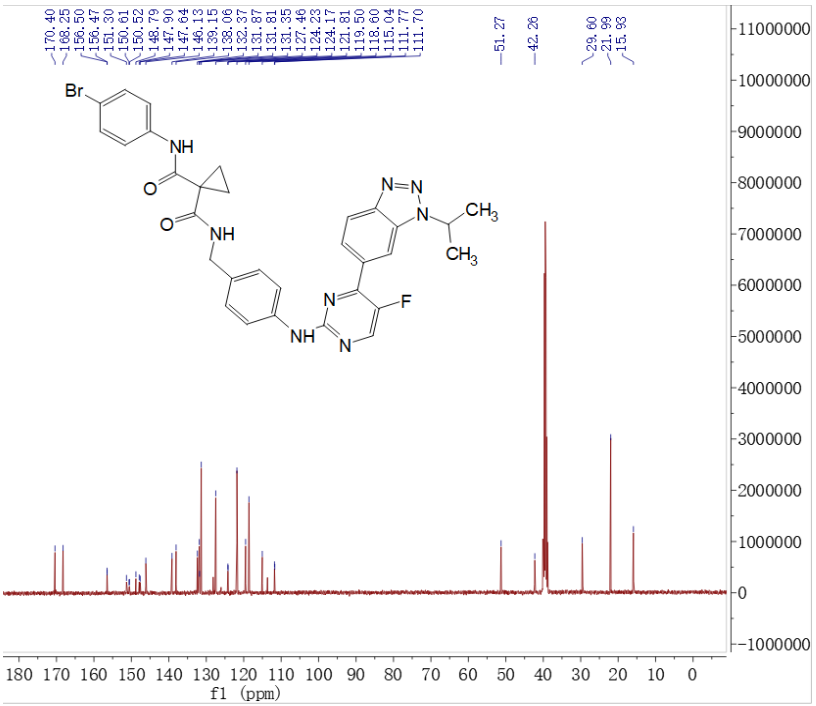

Figure S10. ^{1}H and ^{13}C spectra of compound 12G.

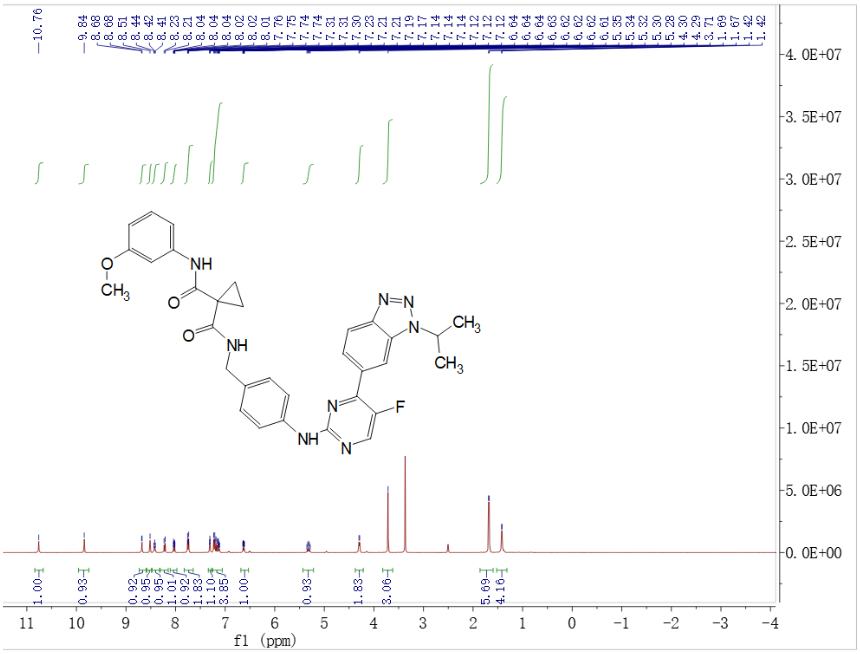

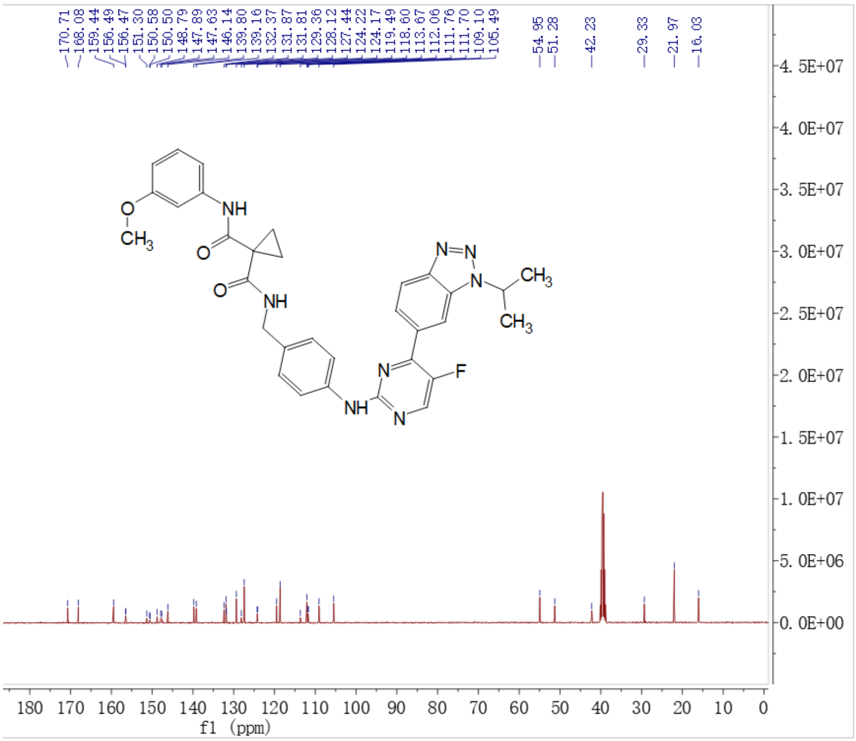

Figure S11. ^{1}H and ^{13}C spectra of compound 12H.

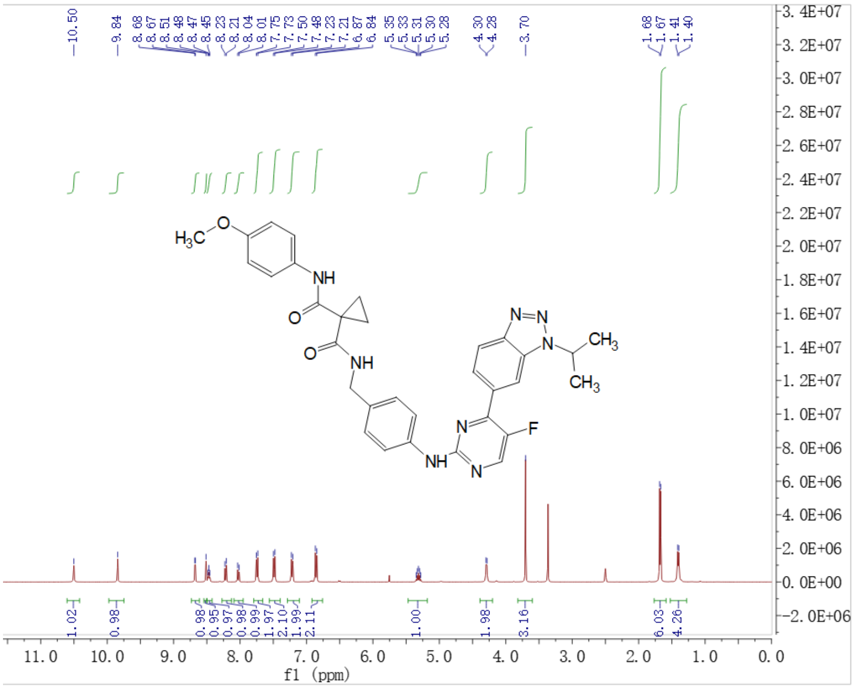

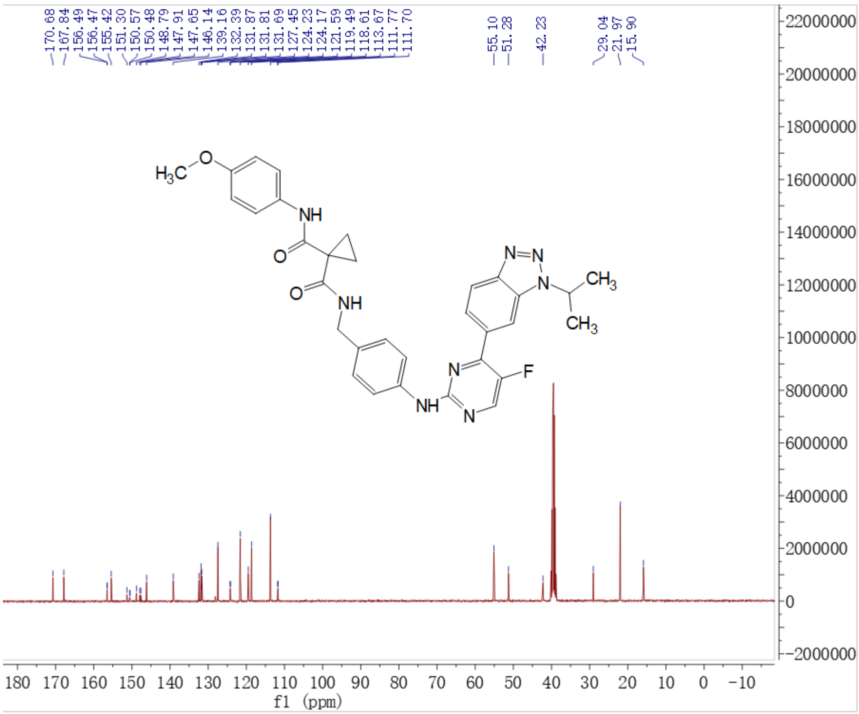

Figure S12. ^{1}H and ^{13}C spectra of compound 12I.

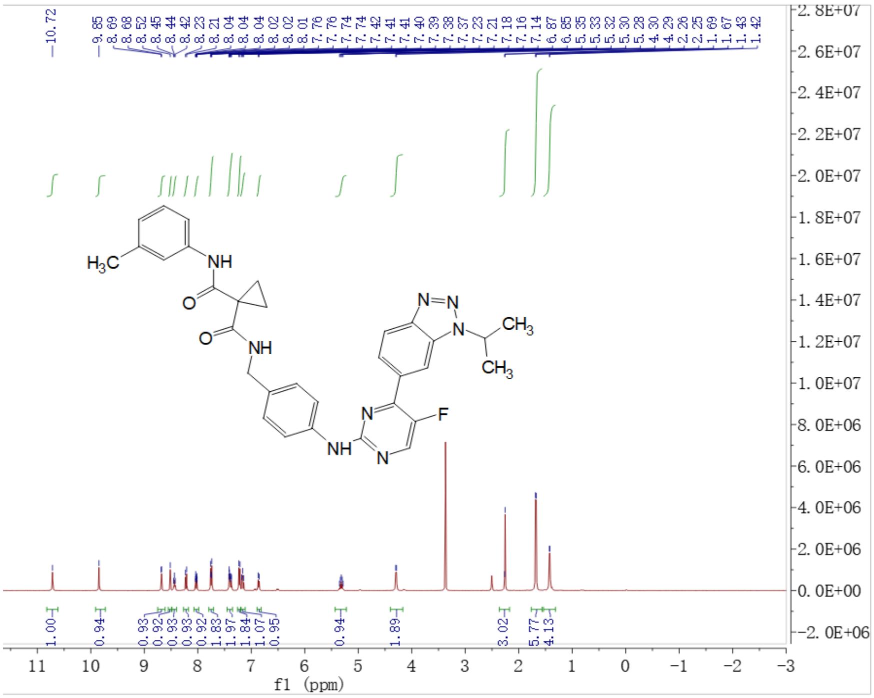

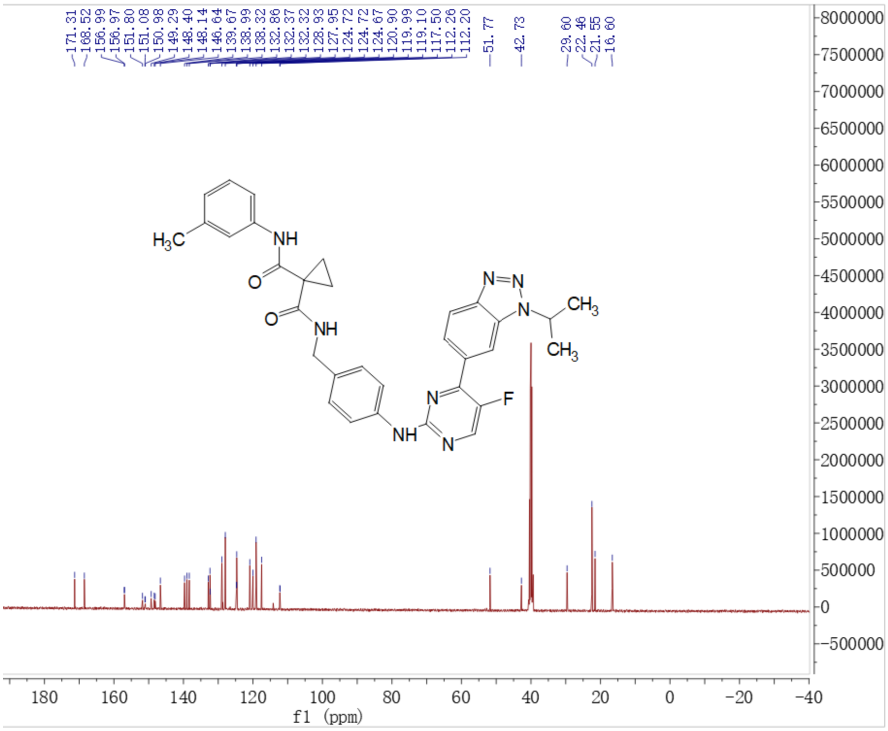

Figure S13. ^{1}H and ^{13}C spectra of compound 12J.

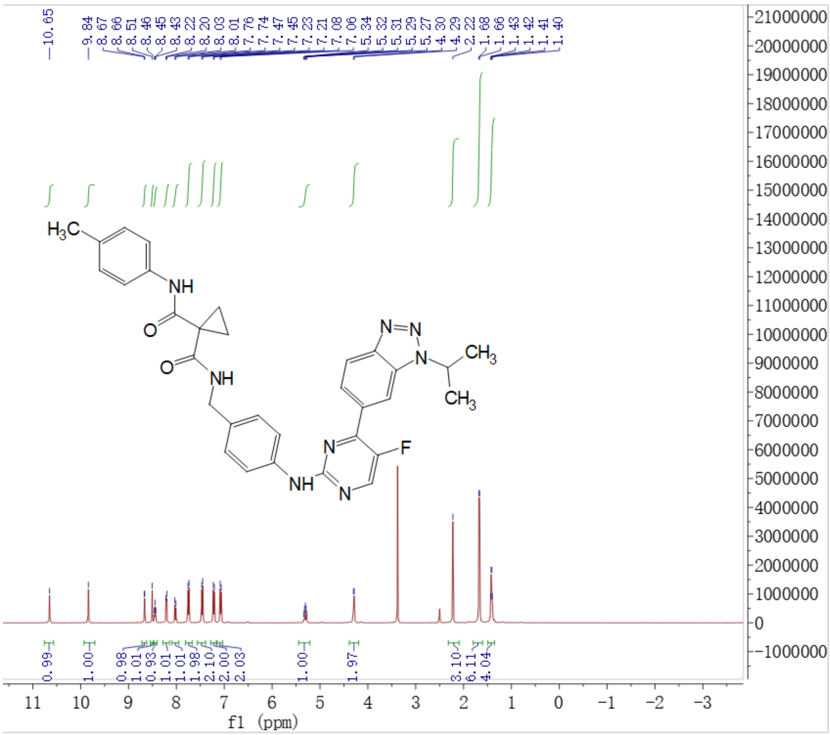

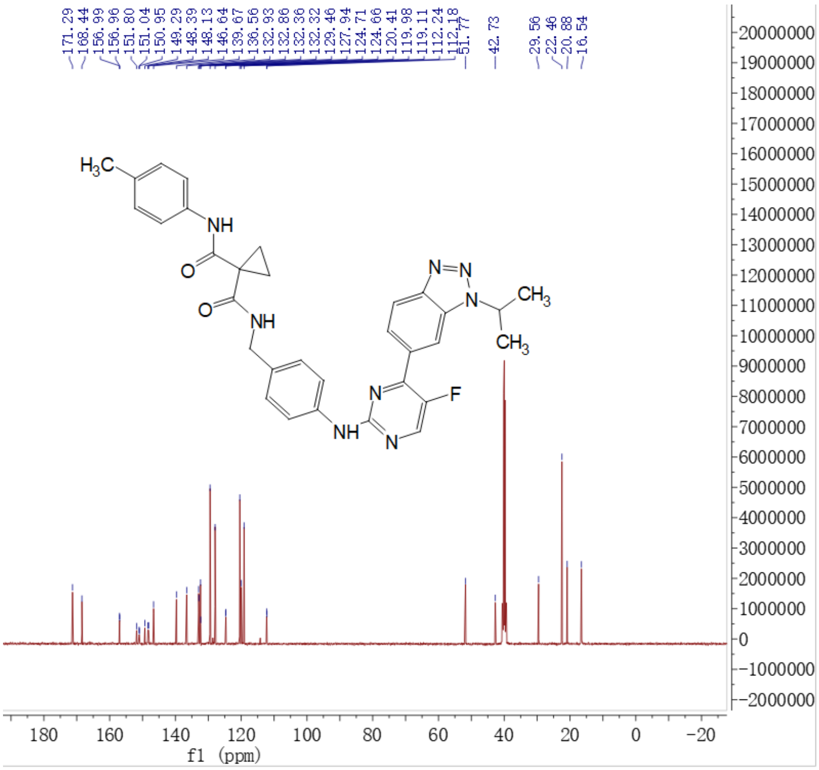

Figure S14. ^{1}H and ^{13}C spectra of compound 12K.

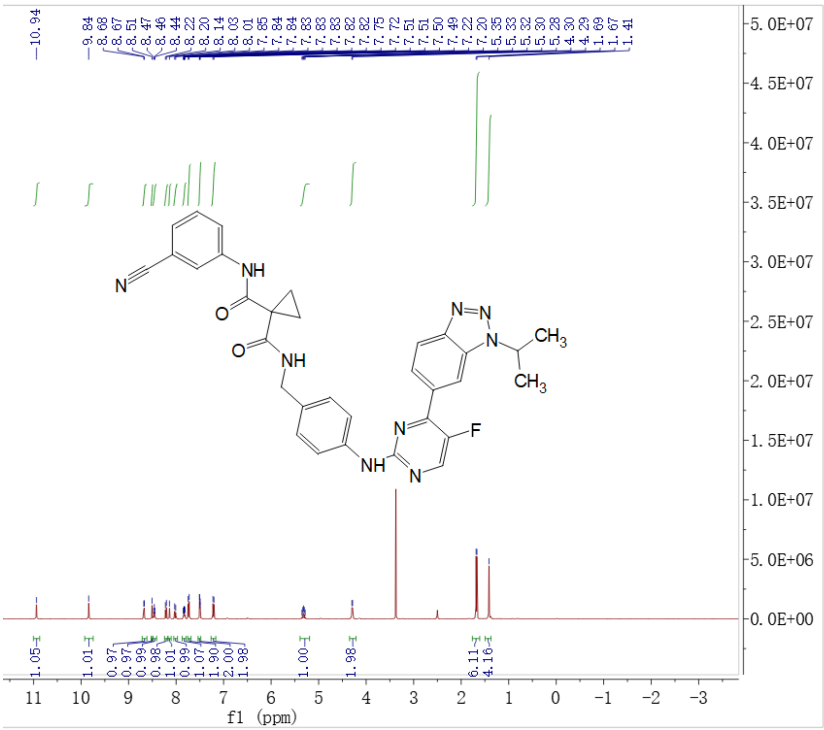

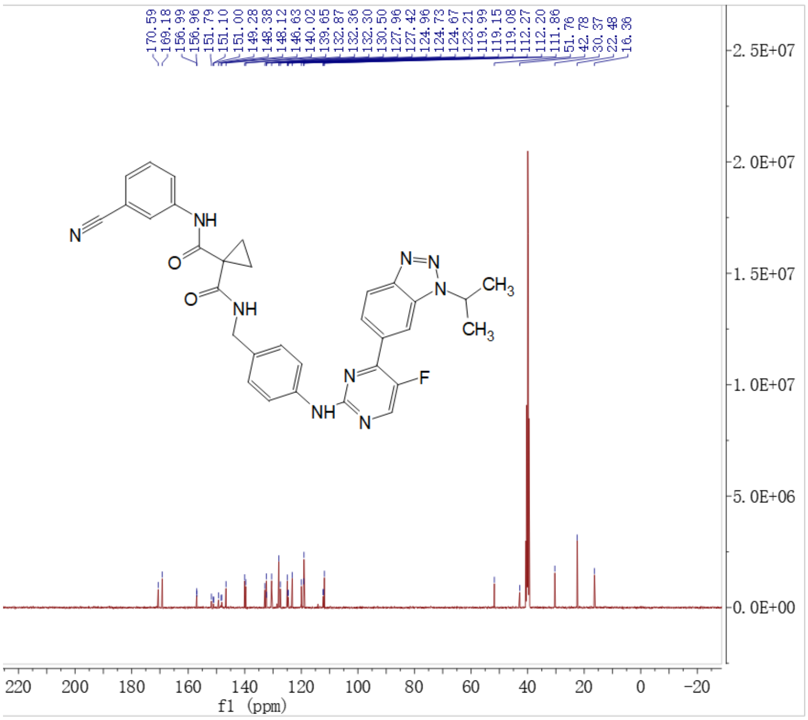

Figure S15. ^{1}H and ^{13}C spectra of compound 12L.

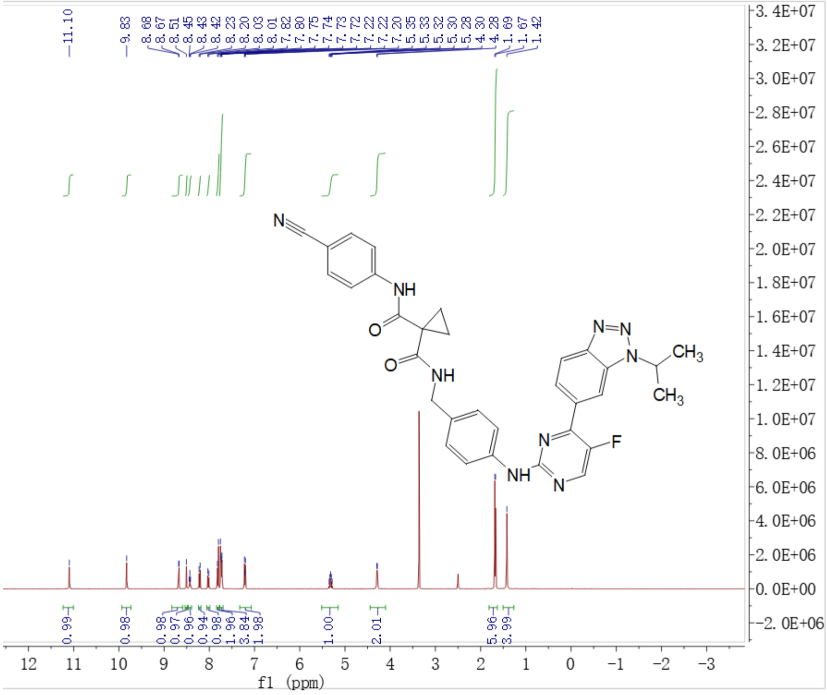

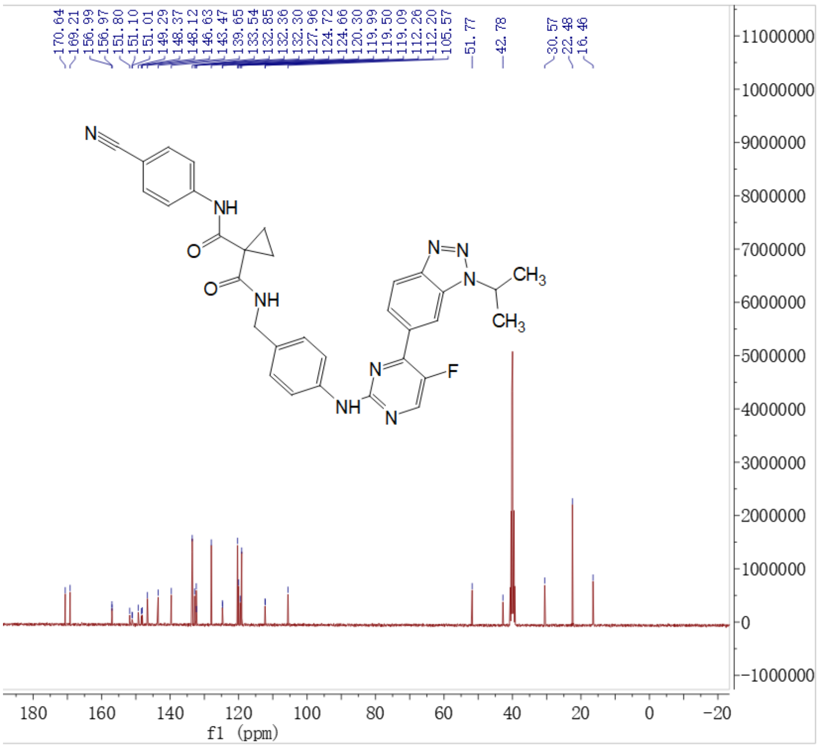

Figure S16. ^{1}H and ^{13}C spectra of compound 12M.

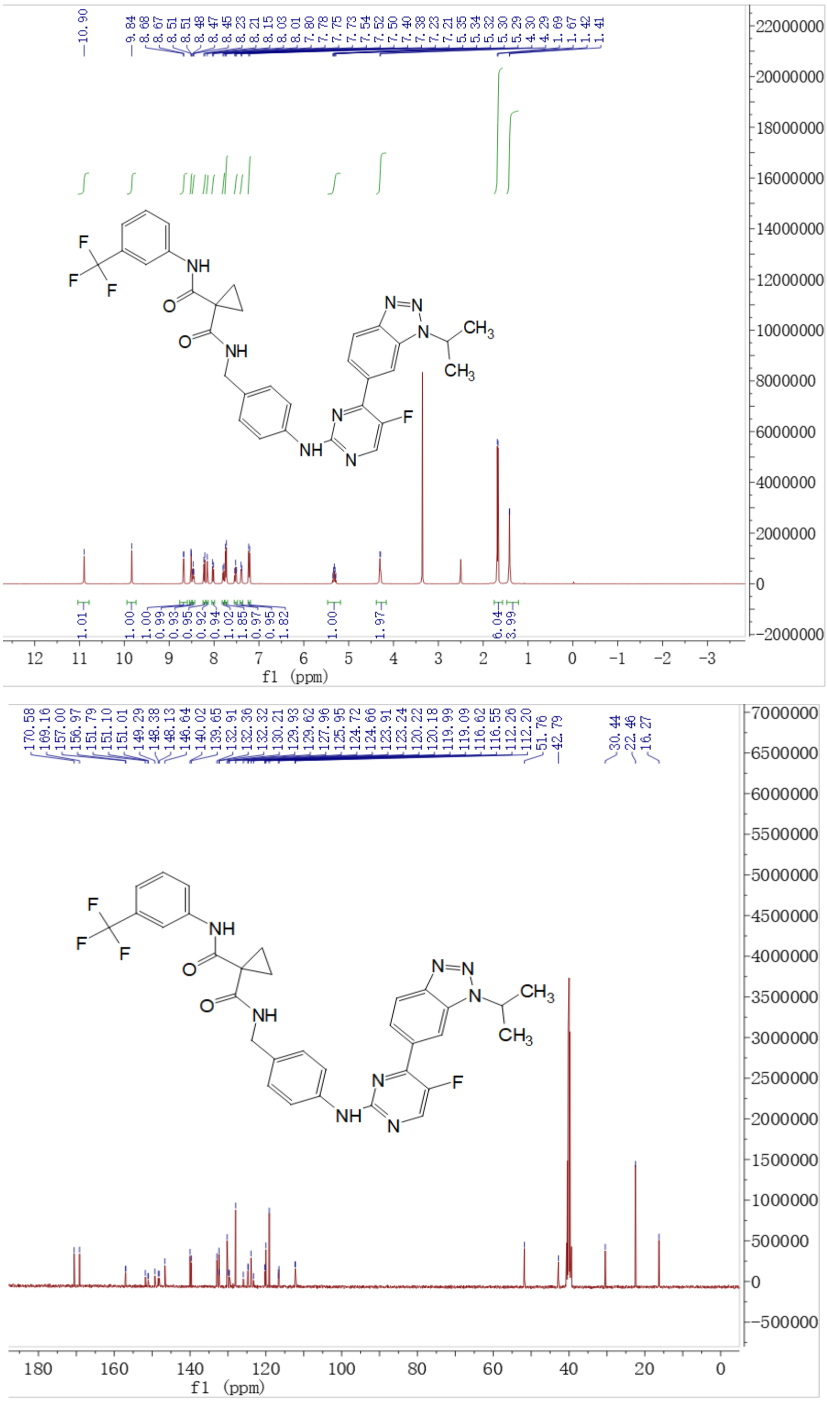

Figure S17. ^{1}H and ^{13}C spectra of compound 12N.

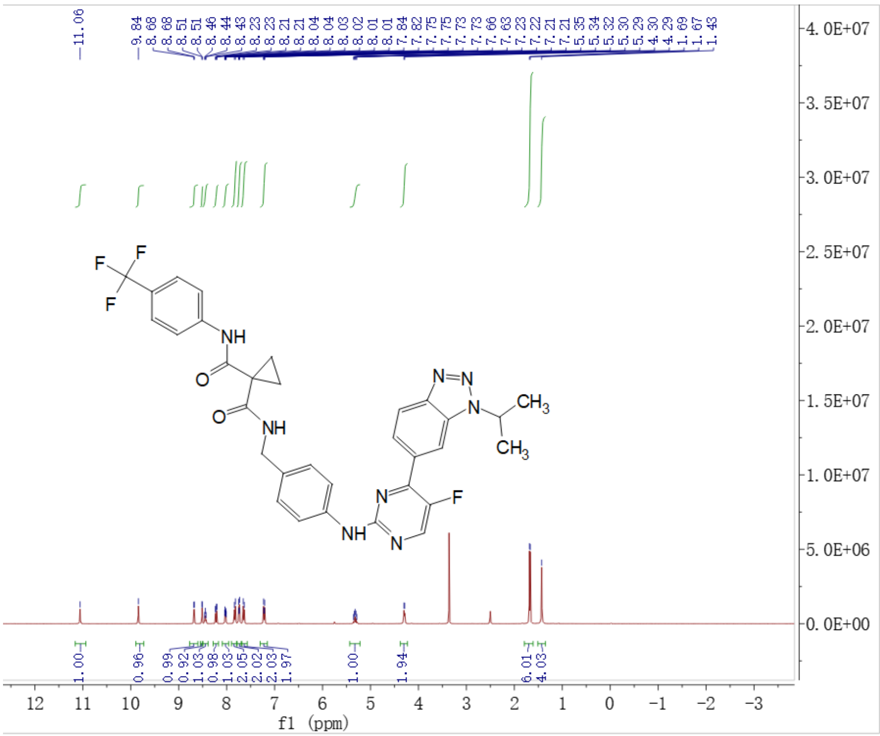

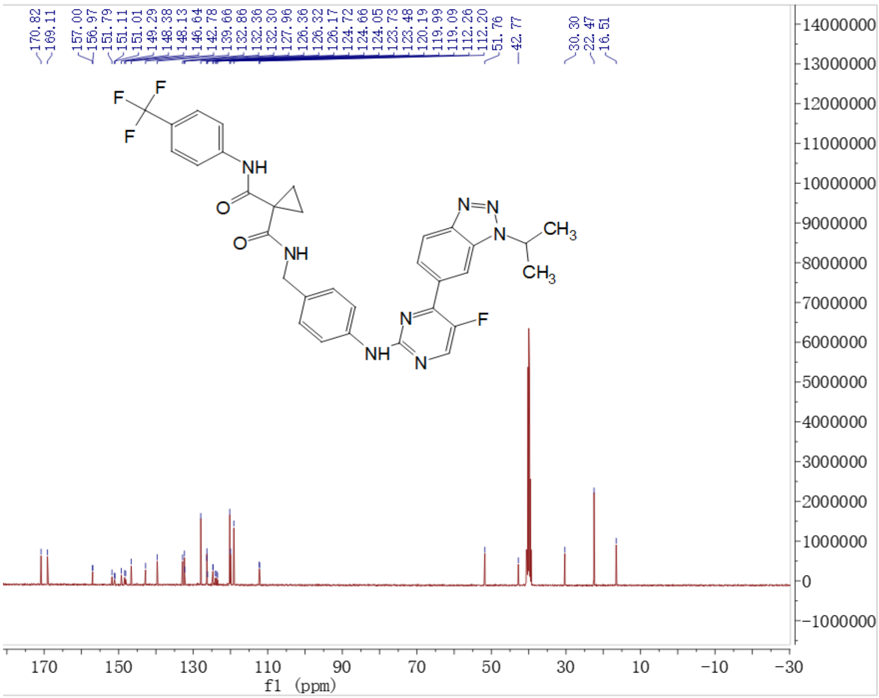

Figure S18. ^{1}H and ^{13}C spectra of compound 12O.

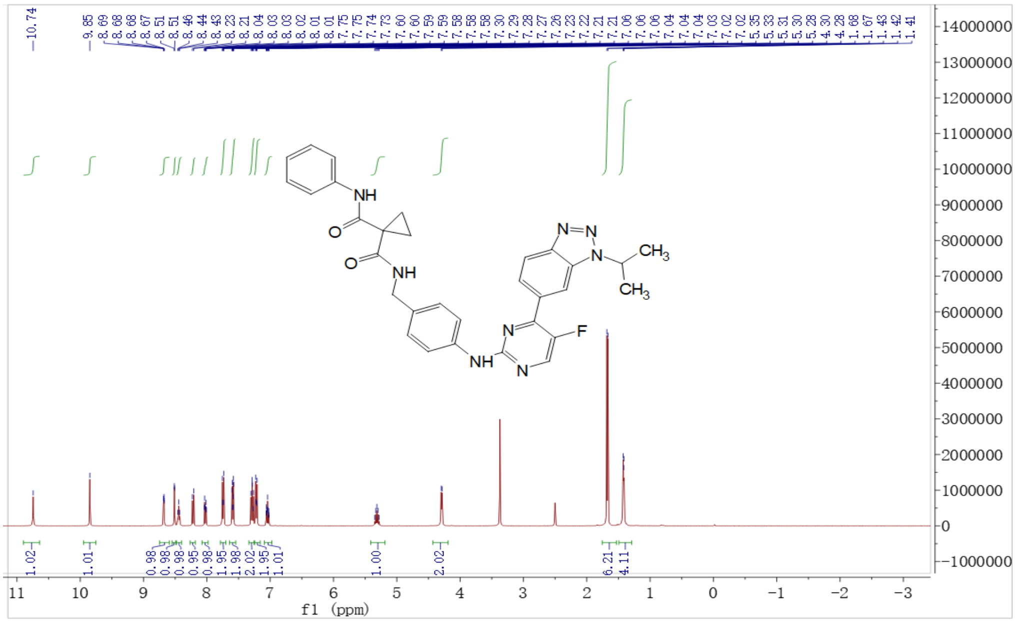

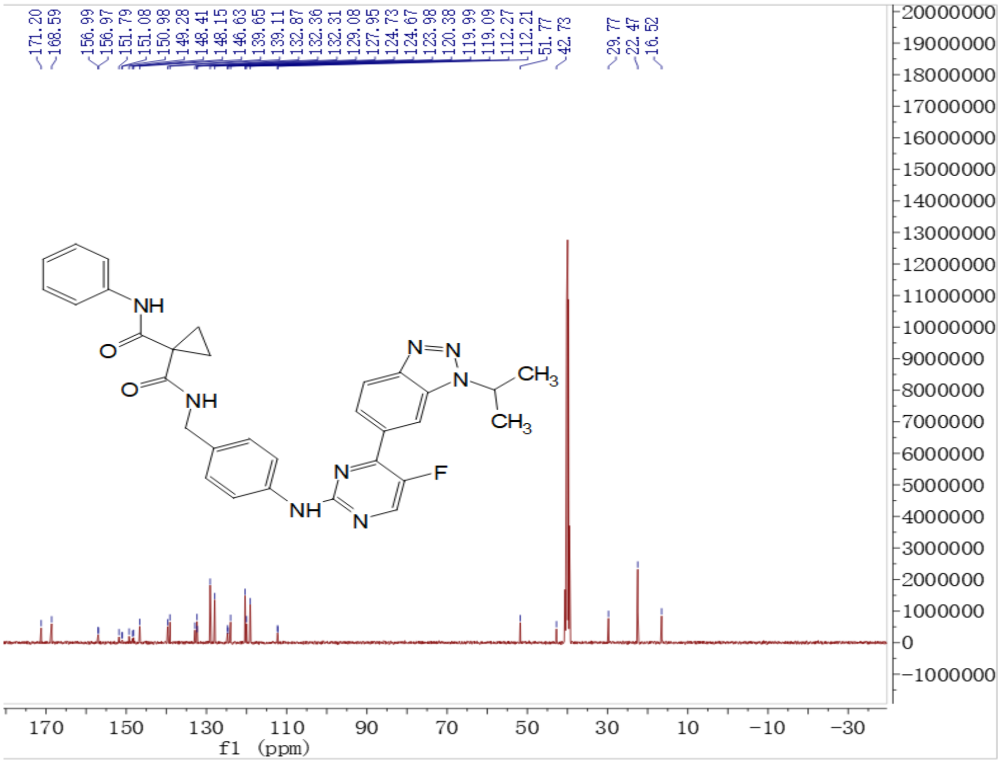

Figure S19. ^{1}H and ^{13}C spectra of compound 12P.

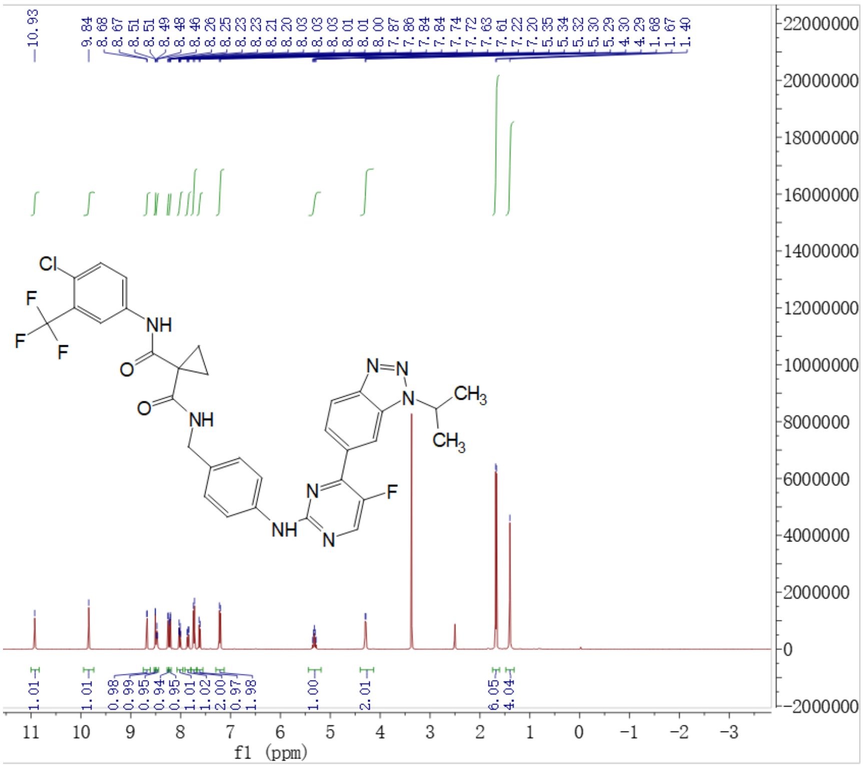

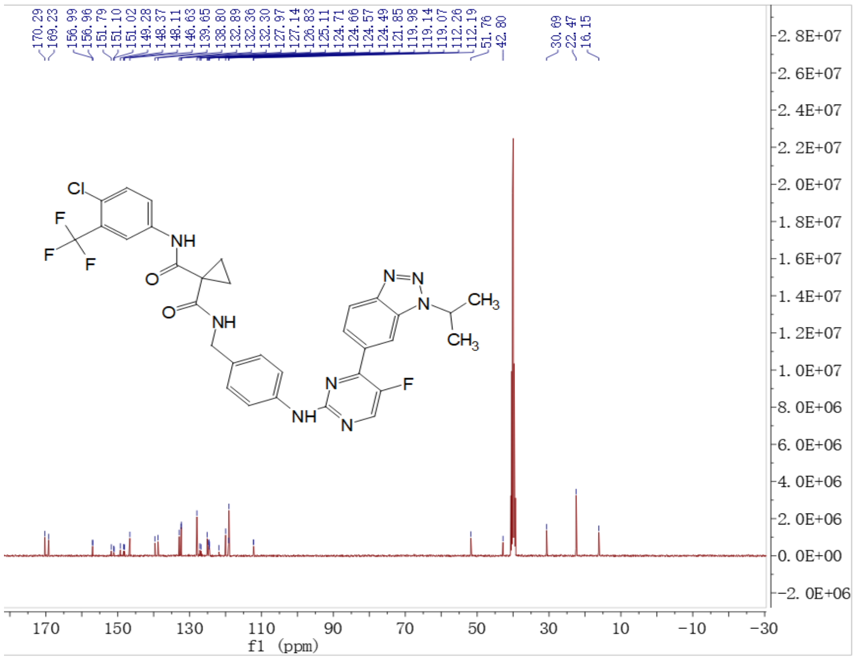

Supplement: Supplementary file 1 [file molecules-25-05199-s001.pdf]
